# Transcutaneous canine breast cancer detection in Tunisia: a pilot study

**DOI:** 10.1186/s12885-023-11599-3

**Published:** 2024-01-30

**Authors:** Imtinene Belaid, Mohamed Fedy Baya, Saif Ben Ayed, Ali Ben Ayed, Jihen Maatoug, Nawel Zommit, Mohamed Anas Trabelsi, Noureddine Ben Chida, Hedi Khairi, Leila Ben Fatma, Imene Chabchoub, Nouha Ammar, Rym Bourigua, Makrem Hochlaf, Faten Ezzaari, Slim Ben Ahmed

**Affiliations:** 1 Faculté de Médecine de Sousse, Farhat Hached University Hospital, Department of Medical Oncology, Association de Recherche et d’Information sur le Cancer du Centre Tunisien (ARIC), Université de Sousse, 4000 Sousse, Tunisia; 2 K9 Dog Security and Training Center, Sousse, Tunisia; 3 Faculté de Médecine de Sousse, Farhat Hached University Hospital, Department of Epidemiology, Université de Sousse, 4000 Sousse, Tunisia; 4 Veterinary Clinic, 4054 Bouhsina, Sahloul Tunisia; 5 National School of Veterinary Medicine, Veterinary Research Institute, Tunis, Tunisia; 6 Faculté de Médecine de Sousse, Farhat Hached University Hospital, Department of Gynecology, Université de Sousse, 4000 Sousse, Tunisia

**Keywords:** Cancer, Diagnosis, Smell, Working dogs, Cancer screening test

## Abstract

**Background:**

Breast cancer in Tunisia is often diagnosed at a late stage with long delay in time to consultation and to diagnosis.The aim of this study is to estimate the sensitivity and specificity of the transcutaneous breast cancer detection by canine olfactionin Tunisian women and to identify the potential confounding factors.

**Methods:**

This is a diagnostic case control study that took place from October 2021 to November 2022 in the Department of Medical Oncology at the University Hospital Farhat Hached of Sousse and in the security and training dog center located in Sousse (K9 Dog Center Security & Training). A two-year-old male Belgian Malinois was trained to detect breast cancer on skin secretion samples in compresses that had been worn overnight by women on their breast and then a double-blind testing was performed. There was no contact between women and the dog. From the mentioned responses of the dog, four parameters were calculated: sensitivity, specificity, Positive Predictive Value (PPV) and Negative Predictive Value (NPV).

**Results:**

Two hundred women were included in this trial: 100 breast cancer (BC) patients recruited from Farhat Hached University Hospital of Sousse and 100 healthy volunteers (HV).The calculated sensitivity was 84% (95% CI 78–89%) and the calculated specificity was 81% (95% CI 75–86%). The calculated predictive values were: PPV = 83,51% (95% CI 78,37–88,65%) and NPV = 81,55% (95% CI 76.17–86.93%). In the multivariate study, only four confounding factors of test’s sensitivity were retained: age (OR = 1.210 [95% CI = 1.085–1.349]; *p* = 0.001), history of diabetes(OR = 0.017 [95% CI = 0.001–0.228]; p = 0.002), sampling at hospital (OR = 0.010 [95% CI = 0.003–0.464]; *p* = 0.010) and testing during chemotherapy courses (OR = 0.034 [95% CI = 0.003–0.404]; *p* = 0.007).For test’s specificity, we retained the three following confounding factors: age (OR = 1,104 [95% CI = 1.021–1.195]; *p* = 0.014), history of benign mastopathy (OR = 0.243 [95% CI = 0.074–0.805]; *p* = 0.021)and history of arterial hypertension (OR = 0.194 [95% CI = 0.053–0.707]; *p* = 0.013).

**Conclusion:**

This is a pilot study that opens new avenues in developing a reliable cancer diagnostic tool that integrates the dog's olfactory ability to detect breast cancer using a transcutaneous sampling method. It could be a pre-test to select patients who are eligible to a screening mammogram, especially in low-income countries where there is no national mammography screening program.

**Pactr.org identifier:**

PACTR202201864472288, registration date 11/01/2022.

## Background

Breast cancer is a public health problem in the world and in Tunisia due to its frequency and severity. It is the first cancer among Tunisian women and accounts for about 34.5% of female cancers [1]. Improving the prognosis of breast cancer requires early diagnosis by mammography screening. In Tunisia, regular (annual or every 2 years) mammography is recommended in women aged 45–74 years, according to the National Authority for Assessment and Accreditation in Healthcare (INEAS) recommendations. However, diagnosis is rarely made by a screening mammogram [2] and breast cancer is often diagnosed at a late stage with long delay in time to consultation and to diagnosis [3].

The late diagnosis of breast cancer in Tunisia seems to be essentially related to a "patient delay" that corresponds to a delay in consultation for women with poor socio-economic conditions and who live far from radiology centers in medical deserts, facing difficulties to access mammograms [4, 5].

Ideally, an accessible, easy-to-use, non-invasive and reliable screening test should allow select women who are eligible to a screening mammogram.

The canine olfactory system appears to be not only able to detect certain substances in a very small proportion, on the order of a few parts per trillion but it is also able to discriminate between complex chemical mixtures found in human body fluids (urine, breath, sweat) [6]. Screening and early diagnosis by canine detection would have many advantages including simplicity, safety of the test, rapidity of results, accessibility and low cost.

Dogs may sniff out cancer-specific Volatile organic compounds (VOC) in human biological samples [7]. In the last two decades, several studies reported the ability of dogs to detect different types of cancer by smelling [8–12]. Studies published on cancer detection by canine olfaction from a urine sample showed promising results [8, 13]. In 2017, Pirrone F et al. highlighted the scientific reports testing canine olfaction to detect cancer, dividing them according to the cancer’s primary site [14].

The only proof-of-concept study using canine detection of breast cancer by sweat was published by Thuleau et al. in 2019 [15]. Two dogs were trained to detect VOC of skin secretion samples on compresses that had been worn overnight by women on their breast. Eighty-seven women were included: 51 healthy volunteers and 36 breast cancer patients. These dogs recognized 90.3% of skin samples from breast cancer patients. The clinical trial phase was launched in 2020 by the Curie Institute in Paris (ClinicalTrials.gov Identifier: NCT04217109). The results are not yet published. Based on the promising results of Thuleau et al. proof-of-concept study [15] and the feasibility of this test in our region, we decided in our project (Pactr.org Identifier: PACTR202201864472288, registration date 11/01/2022) to estimate the sensitivity and specificity of the transcutaneous breast cancer detection by canine olfaction in Tunisian women and to identify the potential confounding factors correlated with the sensitivity and specificity of this test.

## Methods

This is a diagnostic test study that took place from October 2021 to November 2022 in the Department of Medical Oncology at the University Hospital Farhat Hached of Sousse and in the security and training dog center located in Sousse (K9 Dog Center Security & Training). The dog trainer was graduated from the Royal Dutch Police Dog Association (KNPV) which trains scent dogs (police, search and rescue). The study is reported in accordance with ARRIVE guidelines [16].

### Inclusion, non-inclusion and exclusion criteria

Two hundred women were included in this trial: 100 breast cancer (BC) patients and 100 healthy volunteers (HV).

Patients were recruited from Farhat Hached University Hospital of Sousse. We sought patients with any stage disease, especially advanced stage because we suspected that larger tumors might be producing a higher concentration of the chemicals associated with cancer cells and would be more easily detected by the dogs.

#### BC group

They were:

- Women 18 years and older, with a recent biopsy-confirmed conventional diagnosis of breast cancer and not operated yet.
- Women 18 years and older, operated on breast cancer with a recent local relapse of the breast tumor confirmed at histology.
- Patients who signed the informed consent of the study.
- Patients who had no contraindication to simultaneously participate to another research program and there was no exclusion period planned after the study.

We did not include in our study:

- Patients with other types of cancer.
- Patients with a personal history of breast surgery for benign lesion(s) of the concerned breast operated less than 4 weeks before the date of inclusion in this study.
- Patients with a personal history of breast biopsies of the concerned breast less than 4 weeks before inclusion.

BC patients excluded from our study were:

- Patients with a breast skin ulceration, bleeding or infection.
- Patients who had taken antibiotics one week before inclusion in the study.
- Patients with a current viral infection (fever).

In fact, these findings could have potential interference with the study's objectives because we thought they may change the composition of VOCs.

#### HV group

They were women older than 18 years old with a negative screening for breast cancer by mammography and breast ultrasound if the mammography was inconclusive to rule out a breast cancer. Mammograms and breast ultrasound were offered by the ARIC (Association de Recherche et d'Information sur le Cancer du Centre Tunisien) and were performed by the same radiologist. HV group was constituted from the medical and paramedical staff and from their friends and families.

HV who were not included were:

- Women with a personal history of other types of cancer.
- Women with a personal history of breast surgery for benign lesion(s) of the concerned breast operated less than 4 weeks before the date of inclusion in this study.
- Women with a personal history of breast biopsies of the concerned breast less than 4 weeks before inclusion.

**Fig. 1:**
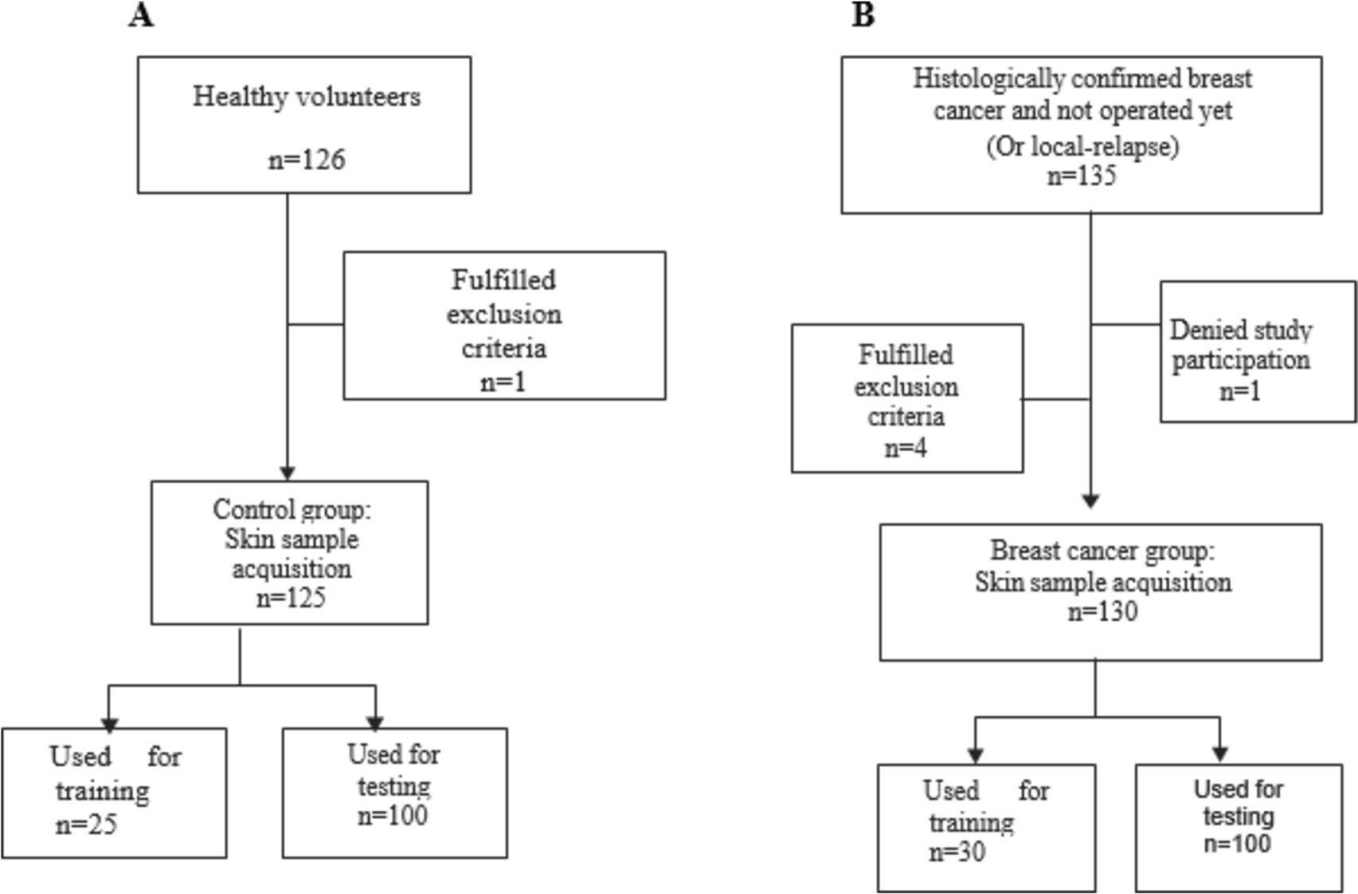
Flowchart diagram of the study for healthy volunteers (**A**) and breast cancer patients (**B**)

Figure 1 represents the flow chart that shows the included and excluded healthy volunteers and breast cancer patients and those whose samples were used for training.

### The study process

**Fig. 2:**
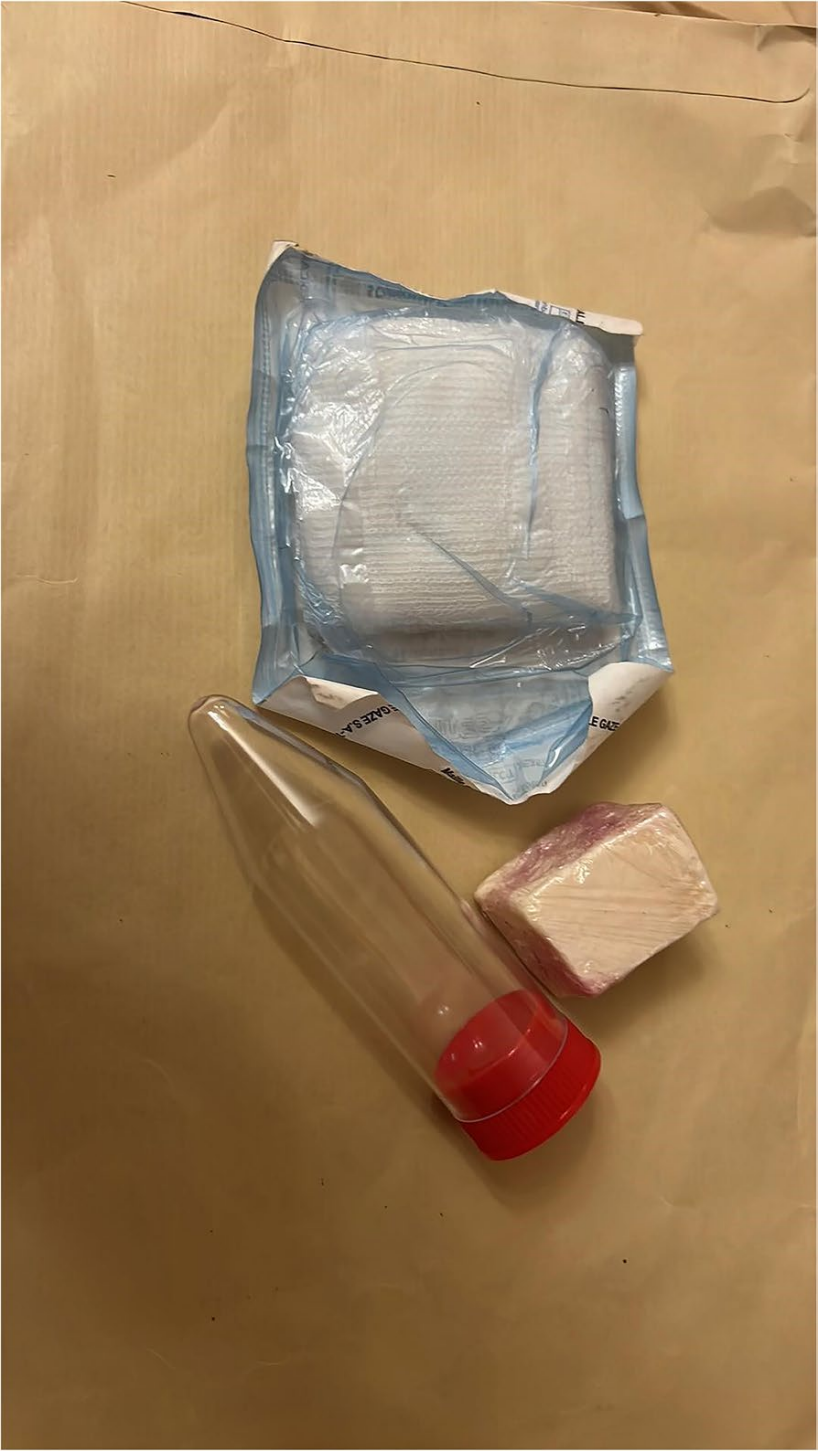
The kit used in our study

Participants were given a kit containing 1 piece of unscented soap, a pack of 5 sterile compress (cotton, 8 × 7 cm) and 1 sterile plastic vial(Fig. 2).The vials used in the study were sterile screw cap conic tube made with polypropylene. They were standardized for all tests. This information was added in the text. Each participant was asked to shower with the given soap then apply a compress in contact with the skin of the concerned breast. HVs positioned a compress on both breasts whereas BC patients positioned a compress on the affected breast. The compress was kept in place during the whole night. For BC patients, this procedure was done on the night prior to surgery or the night prior to the chemotherapy cure.

The compresses, containing skin secretion, once removed in the next morning, were placed in a specific sterile plastic vial and put in brown shade envelops to avoid sun exposure under ambient temperature. Then, they are delivered to the investigating center where they are labeled with a code number with reference to the woman and sent to the laboratory of the K9 Dog Center Security and Training in Sousse.

**Fig. 3:**
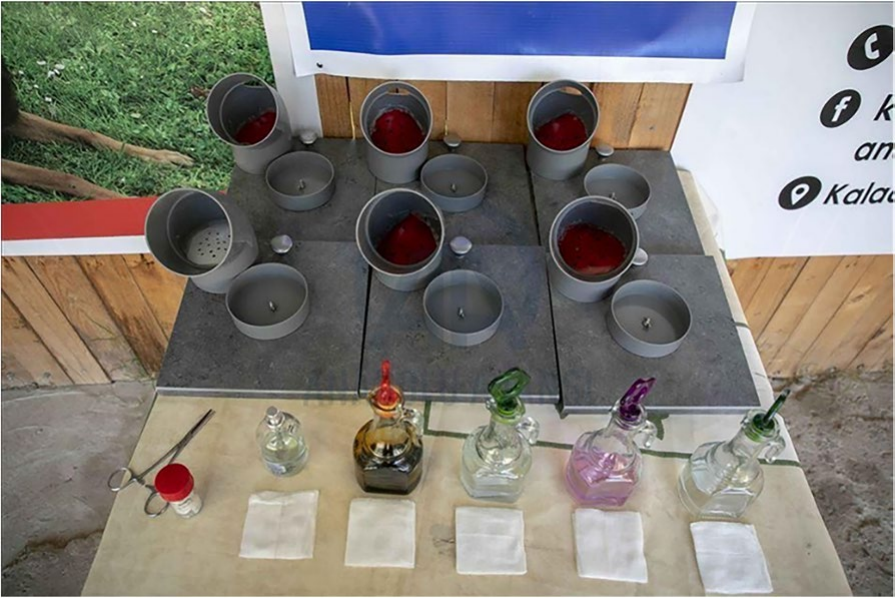
Preparation of olfaction cones

The specimens were packed in jars and kept in a room at ambient temperature. Sample storage time varied between 1 and 30 days. The test took place in a large outdoor kennel with an average ambient temperatures during our study ranged from 15 °C to 35 °C. Five identical cones were used arranged in a straight line spaced 1 m apart. The cones used in the study were made with plastic. In the center of the cones there was only one hole which was filled with a sample coming from either the experimental or control group. The other cones contained various distractor stimuli likely to be encountered in a medical setting (e.g., betadine, sterile compress, alcohol, food) (Fig. 3).

Each box was covered with a metal mesh so that the skin sample can’t be visible or accessible to the dogs other than by olfaction. To prevent cross-contamination, the investigator wore sterile gloves when handling sample vials. At the end of each day, the stations were cleaned with water and vinegar solution.

**Fig. 4:**
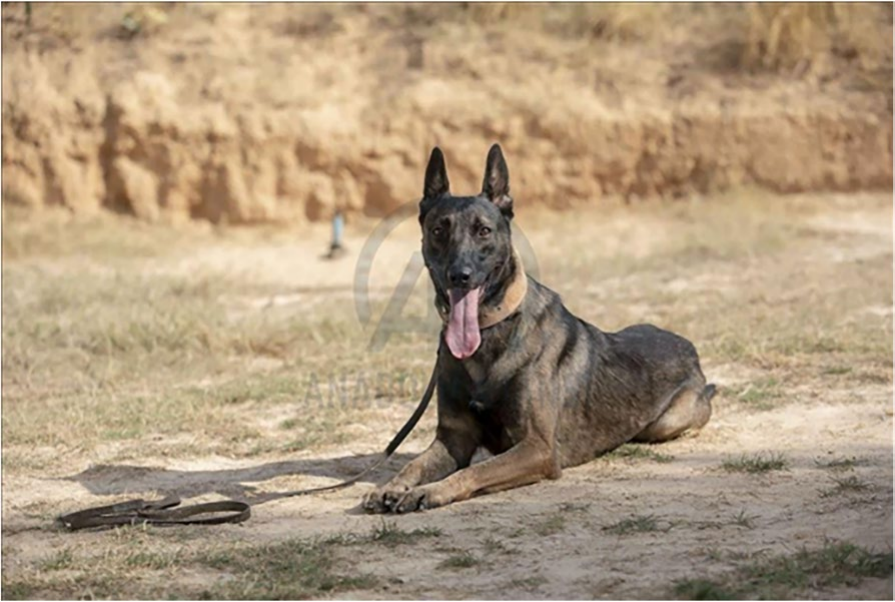
The study dog Roy

Roy, a two-year-old male Belgian Malinois (Fig. 4)was chosen based on its proven success in previous missions.

Training and handling methods were designed in consultation with 2 veterinarians and an independent dog trainer who all approved our methods. The objective was to teach the dogs to discriminate the odors of skin secretion samples taken from women with breast cancer and those who were cancer free. The training process was guided by the design of the study of Mcculosh [9].

Training was performed with of a reward-based approach using the clicker training method. A trainer would simultaneously trigger the clicker device and offer a food snack or ball whenever the dog correctly indicates on the station containing the cancer sample.

The dog training lasted for 5 months, from June 2021 to October 2021, in two training phases with a rest interval of 2 months in between. Under certain weather conditions (such as high temperature and high humidity in summer) the dog sniffing test could not be conducted because the dog could not maintain high levels of concentration.

During the first phase of training, the location of the cancer sample was known by both experimenter and trainer. The target specimen was placed in a random station while the remaining 4 stations were empty. The objective of this phase of training was to teach the dog to indicate on a cancer sample by sitting in front of the station containing the cancer sample. In order to achieve this the dog handler would trigger the clicker signal, order the dog to issue the “sit “command then offer a food or ball reward for the dog.

During the second phase of training, only the experimenter was aware of the location of the target sample. The location of the target specimen was varied over the course of the trials while the dog handler was absent from the training area. Dogs were either tied to a leash or walked free but kept next to the trainer. No limits were imposed on the amount of time the dogs spent sniffing samples and the trainer was given the freedom to encourage the dog to recheck any location an unlimited number of times. However, in this time, the experimenter gave no “sit” command to the dog. Once the study coordinator confirmed that the identification was correct, the clicker was activated and the dog was rewarded as described above. In case of incorrect response, the handler would mildly rebuke the dog by saying “no” and deprive the dog of the reward. The trial was considered complete and the trainer led the dog out of the training area.

The third phase of training was a single-blinded experiment. Dogs were challenged with BC sample and the remaining 4 stations contained various distractor stimuli. The dog had to discriminate between BC samples and control samples.

To encourage the dog to generalize on a common cancer odor, we used skin samples from different donors. BC and HV samples from new donors became available in batches of 5 to 10 at intervals over the training period, and it was sometimes necessary to reuse skin samples from the same donors several times during training.

**Fig. 5:**
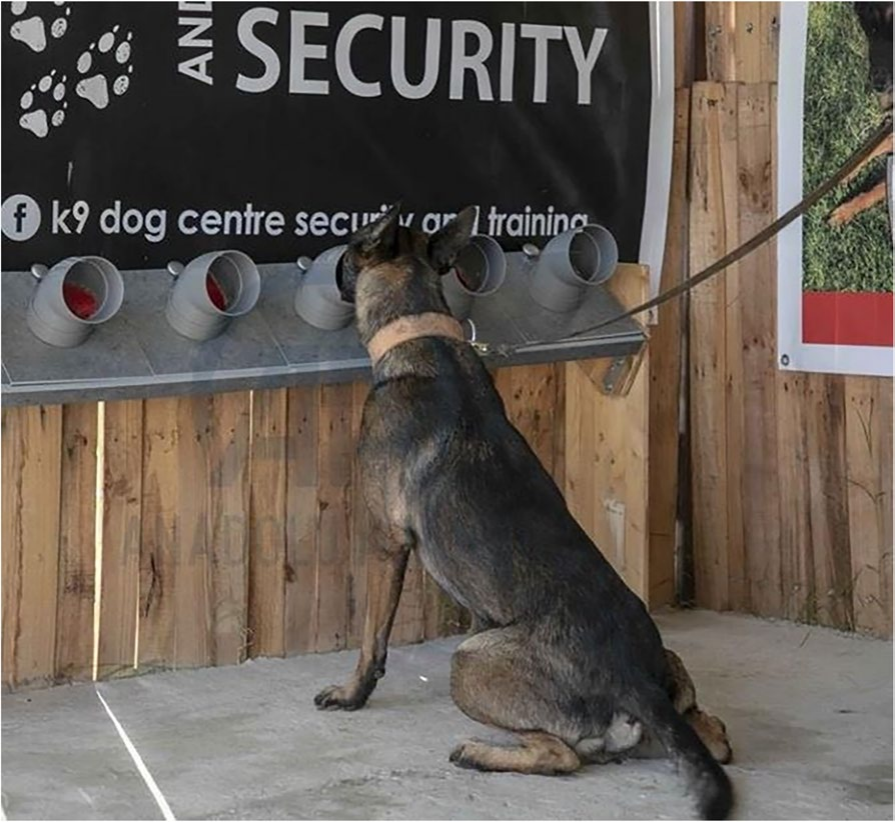
Roy marking a cone containing a positive sample

A successful response was defined as: (i) identification of the target specimen by sitting in front of the station containing a cancer sample (True positive) (Fig. 5), or (ii) sniffing a control sample and not sitting in front of it (True negative).

An incorrect response was defined as: (i) identification of the control specimen as the target specimen (False positive), (ii) sniffing without sitting in front of the station containing a cancer sample (false negative), or (iii) hesitation or unclear behavior on the part of the dog (either false-positive or false-negative depending on whether hesitation was on a cancer or control sample).

The verdict of the test was announced after confirming that the dog did not move spontaneously for three full seconds. If the dog started to move before that time, the test verdict was temporarily suspended. In such cases, assessment was determined when the dog sat in front of the test box without moving for three full seconds.

Training was considered complete by the trainer when the dogs could most of the time detect the breast cancer patient’s sample from among those of 4 control samples.

Double blinding was afterwards introduced in the training. During the entire double-blinded testing phase, we didn’t reuse any of the samples encountered by the dogs during the training phases. All other methods were identical to the single-blinded testing phase, except that we now:

1. placed the target skin sample of interest, whether from patient or healthy volunteers, within the lineup along with four other distractors as described in the first phase.
2. none of the experimenter or the dog handler were aware of the status of the target sample in the lineup to ensure that neither experimenters nor handlers could be giving any clues to the dogs. Since the experimenters now no longer knew the status of the target sample there were no clicker activation or food rewards offered to the dogs. The results were not decoded until the dog had completed all the rounds.

### Data analysis

From the mentioned responses of the dog, four parameters were calculated: sensitivity, specificity, Positive Predictive Value (PPV) and Negative Predictive Value (NPV).

For BC patients, epidemiological, clinical and paraclinical data were collected from medical records of the medical oncology department of Farhat Hached University hospital of Sousse. For HV, only epidemiological variables were studied.

The collected data were entered and analyzed using SPSS version 26 "Statistical Package for Social Sciences" software. Quantitative variables were described by means and Standard Deviations (SD). Qualitative ones were described by numbers and percentages. The Chi2 test was used to compare percentages. When its application conditions were not met, Fisher's exact test was used. Observations with missing data were not considered during comparisons.

A multivariate analysis using a binary logistic regression with a stepwise backward approach was performed. The dependent variables were: “false positive” and "false negative". The "yes" category, coded by the value "1", refers to participants for whom the dog correctly indicated the sample. The "no" category, coded by the value "0", refers to participants for whom the dog failed to correctly indicate the sample. Overall agreement and agreement between the two measurements. were assessed using the Mac Nemar test and Kappa statistic (K) considering values < 0 as indicating no agreement, 0–0.20 as slight, 0.21–0.40 as fair, 0.41–0.60 as moderate, 0.61–0.80 as substantial, and 0.81–1 as almost perfect agreement [17]. All the explanatory variables which were associated with these dependent variables during the univariate analysis with a significance degree < 0.2 were included in the initial multivariate analysis models.

Observations with missing data were not included when performing the different regression models. Results of the regression model were expressed as odds ratios (ORs) with confidence interval (CI) of 95%. All statistical tests were two-tailed, and *p*-values < 0.05 were considered statistically significant.

### Ethics

Our protocol and patient recruitment materials were approved by an institutional Ethics Committee (The Research Ethics Committee of Farhat Hached Hospital).All subjects provided their oral and written informed consent to participation in the study. The objectives and implications of the work were well explained to them. To protect the confidentiality of the patients, the vials were sent in an anonymous way to the laboratory of the K9 Dog Center Security and Training in Sousse.

## Results

### Study population characteristics

Twenty-nine patients performed sampling during their hospitalization.

All the participants’ epidemiological characteristics and past medical history are summarized in Table 1.

**Table 1:** Characteristics of the 200 participants

Variables	Overall (*n* = 200)	Breast cancer group (*n* = 100)	Control group (*n* = 100)
Female, n(%)	200 (100)	100(50)	100(50)
Age, (years), median [interquartile range]	51,82 [26; 81]	53,47 [26;81]	50,17[29;66]
Menopausal, n (%)	107(53)	56(56)	51(51)
Most frequently reported past or current diseases			
Hypertension,n (%)	60(30)	32(5)	28 (28)
Diabetes, n (%)	64(32)	32(4)	34 (34)
Dyslipidemia, n (%)	60 (30)	34(3)	28 (28)
Mastopathy, n (%)		NA	23 (23)
Current treatments			
Oral anti-diabetic, n (%)	51(25)	23 (23)	28(19)
Insulin, n (%)	29(14)	16 (16)	13(13)
Anti-hypertension, n (%)	56(28)	30(30)	26 (26)
Anti-dyslipidemic, n (%)	54(27)	30 (30)	24(24)

*NA* Non applicable

The mean age of the HV patients was 50.17 ± 8.25 years. The majority (51%) were post-menopausal. A history of diabetes was mentioned in 34 women (34%). 28 had a history of hypertension. Twenty-eight women had a history of dyslipidemia. Twenty-three women had a benign mastopathy.

The mean age of BC patients was 53.47 ± 12.43 years. The majority (56%) were post-menopausal. BC patients who had a medical history represented 39% of the study population (*n* = 39). A history of diabetes was mentioned in 32 patients (32%). 32 had a history of hypertension and 34 had a history of dyslipidemia.

BC anatomo-clinical and therapeutic characteristics are summarized in Table 2.

**Table 2:** Anatomo-clinical and therapeutic characteristics of breast cancer group

**Characteristics *(n* = *100)***	
Mean age (years)	50.17 ± 8.25
	**Number of patients (%)**
Clinical T stage	
T0-Tis	4 (4)
T1	19 (19)
T2	27 (27)
T3	16 (16)
T4	34 (34)
T4a	3 (3)
T4b	15 (15)
T4c	6 (6)
T4d	10 (10)
Clinical lymph nodes N stage	
N0	47 (47)
N1	33 (33)
N2	16 (16)
N3	4 (4)
Mammography American college of radiology (ACR) classification	
0	0(0)
1	0 (0)
2	0 (0)
3	2(2)
4	33(33)
5	64 (64)
Non specified	1 (1)
Molecular subtypes	
Triple negative	16.2 (16.2)
Luminal A	14.1 (14.1)
Luminal B HER2 negative	38.4 (38.4)
Luminal B HER2 positive	25.3 (25.3)
HER2 overexpression	6.1 (6.1)
Timing between testing and chemotherapy	
Before starting chemotherapy courses	53 (53)
During chemotherapy courses	39 (39)
After completion of chemotherapy courses	8 (8)
Testing for de novo breast cancer or local relapse	
De novo	91 (91)
Local relapse	9 (9)

Among 99 patients for whom clinical size was specified, the mean clinical size at diagnosis was44.5 mm ± 29.9 with extremes ranging from 0 to 160 mm. Tumors classified as T2 were the most frequent (27%). The majority of our patients (47%) did not have a nodal involvement. Twenty patients were metastatic de novo.

The most frequent histological subtype was invasive ductal carcinoma, representing 92% of the breast cancers, followed by invasive lobular carcinoma, which accounted for 6%. In 2% of cases, patients had a mixed subtype. The majority of patients had luminal B HER2-negative tumors (38.4%). 53 patients had undergone the test before the start of any chemotherapy course. Only nine tests were performed on local relapses.

### Test results

During the double-blind testing, the calculated sensitivity was 84% (95% CI 78–89%) and the calculated specificity was 81% (95% CI 75–86%).

Overall agreement was measured between transcutaneous test results and mammography results. We found an agreement between the two results in 165 women (82.5%) whereas disagreement was found in 35 women (17, 5%) (Kappa(K) = 0,65; *p* = 0, 736).

PPV was 81, 55% (95% CI 76.17–86.93%). NPV was 83, 51% (95% CI 78, 37–88,65%).

In Table 3, we present the results of the double-blind test, where the dogs' indications for cancer patient samples and control samples are detailed. The numbers include 84 true positives, indicating correct cancer sample detection; 16 false negatives, indicating missed cancer sample detection; 19 false positives, indicating incorrect indications for control samples; and 81 true negatives, indicating correct control sample detection.

**Table 3:** Results of double-blind tests

	Sample	
Dog indication	Cancer	Control
Positive	84	19
Negative	16	81

Potential confounding factors were analyzed. We looked for associations between the test results and the socio- demographic, clinical and therapeutic characteristics of the two groups of women.

Confounding factors were studied in BC group and those influencing specificity were studied the HV group.

The test's specificity according to the characteristics of healthy volunteers are shown in Table 4. We have identified significant factors that affect the test's specificity. Notably, age (*p* = 0.047) and benign mastopathy (*p* = 0.028) have been found to have a significant impact on the test's specificity.

**Table 4:** Test's specificity according to healthy volunteers’ characteristics

Factors	Status	Specificity (%)	*p* value
Age	–	–	**0,047**
Menopausal status	Non-menopausal	79,6	0,725
	Post-menopausal	82,4	
Diabetes	No	83,3	0,407
	Yes	76,5	
Arterial Hypertension	No	84,7	0,128
	Yes	71,4	
Dyslipidemia	No	84,9	0,099
	Yes	70,4	
Benign Mastopathy	No	85,7	**0,028**
	Yes	65,2	

The test's sensitivity according to breast cancer characteristics are summarized in Table 5. We have identified significant factors that affect the test's sensitivity. Notably, older and menopausal women demonstrated better sensitivity rates. Conversely, women with comorbidities such as diabetes, arterial hypertension, and dyslipidemia, as well as those undergoing chemotherapy, exhibited lower sensitivity rates. Additionally, we observed that sampling at home was correlated with better sensitivity rates.

**Table 5:** Test's sensitivity according to breast cancer patients’ characteristics

Factors	Status	sensitivity(%)	*P* Value
Age	–	–	**0,033**
Menopausal status	Non-menopausal	79,6	**0,03**
	Post-menopausal	82,4	
Diabetes	No	83,3	0,023
	Yes	76,5	
Arterial Hypertension	No	84,7	**0,023**
	Yes	71,4	
Dyslipidemia	No	84,9	**0,009**
	Yes	70,4	
Sampling Area	Home	85,7	**0,015**
	Hospital	65,2	
Multifocality	No	85,8	0,459
	Yes	79,2	
Tumor size	Small	80	0,275
	Large	88	
Metastatic status	Non-metastatic	82,5	0,413
	Metastatic	90	
Stage	Early	76,7	0,086
	Advanced	89,5	
Histological subtype	Invasive ductal carcinoma	86	0,540
	Invasive lobular carcinoma	57,1	
Grade	SBR I—II	82,8	0,666
	SBR III	89,5	
Hormone receptors	Negative	80	0,079
	Positive	85,1	
Her2neu status	Negative	88,4	0,084
	Amplified	74,2	
Delay from chemotherapy	Far from CT courses	91,4	**0,018**
	During CT courses	73,8	

In the multivariate study, three confounding factors for test’s specificitywere retained (Table 6):

- age (OR = 1,104 [95% CI = 1.021–1.195]; *p* = 0.014).
- history of benign mastopathy (OR = 0,243 [95% CI = 0.074–0.805]; *p* = 0.021).
- history of hypertension (OR = 0.013 [95% CI = 0.053–0.707]; *p* = 0.013).

**Table 6:** Factors influencing test specificity in multivariate analysis

Factors	p	OR	Confidence interval 95%	
			Inferior	Superior
Age	**0,014**	**1,104**	1,021	1,195
Benign mastopathy	**0,021**	**0,243**	0,074	0,805
Arterial hypertension	**0,013**	**0,194**	0,053	0,707

In the multivariate study, four confounding factors for test’s *sensitivity*were retained (Table 7):

- age (OR = 1.210[95% CI = 1.085–1.349]; *p* = 0.001).
- history of diabetes (OR = 0.017 [95% CI = 0.001–0.228]; *p* = 0.002).
- sampling area (OR = 0.039 [95% CI = 0.003–0.464]; *p* = 0.010).
- Delay between chemotherapy and test (OR = 0.034 [95% CI = 0.003–0.404];*p* = 0.007).

**Table 7:** Factors influencing test sensitivity in multivariate analysis

Factors	p	OR	Confidence interval 95%	
			Inferior	Superior
Age	**0,001**	**1,210**	1,085	1,349
Diabetes	**0,002**	**0,017**	0,001	0,228
Sampling at hospital	**0,010**	**0,039**	0,003	0,464
Test during CT	**0,007**	**0,034**	0,003	0,404

## Discussion

In our study, the trained dog successfully detected and distinguished skin secretion samples of patients with breast cancer from control group, and 84% (95% CI 78–89%) sensitivity and 81% (95% CI 75–86%) specificity rates could be achieved in the double-blind test series. These results confirm that our test worked and provide convincing evidence that dogs do have the ability to discriminate breast cancer patients from presumptive healthy individuals. The present test result is consistent with previous studies [9, 15]. Thuleau et al. trained two dogs to distinguish, by scent detection, skin secretion samples of breast cancer patients from those of healthy controls. The authors reported a detection sensitivity of 90.3% (Dog 1 had 4/4 correct identification while dog 2 had 21/24 correct identification) on 51 healthy volunteers and 36 breast cancer patients [15].

Table 8 summarizes key studies [8–11, 15, 18–24] that have been published in scientific journals to date on scent cancer detection topic, grouping them together according to the cancer’s primary site of occurrence.

**Table 8:** Summary of previous studies on cancer detection by canines

Cancer type	Study	Odour samples	Detection sensitivity %	Detection specificity %	Percent of correct indications by chance %
**Breast**	Thuleau et al. 2018 [15]	Skin	90,3	––	1/4
	Mcculosh et al. 2006 [9]	Breath	88	98	1/5
	Gordon et al. 2008 [10]	Urine	22	––	1/7
**Ovary**	Horvath et al. 2008 [19]	Tissue	100	97.5	1/4
**Bladder**	Elliker et al. 2014 [22]	Urine	19	73	1/4
	Willis et al. 2004 [8]	Urine	41	––	1/7
	Willis et al. 2011 [18]	Urine	64	56	1/7
**Prostate**	Cornu et al. 2011 [11]	Urine	91	91	1/6
	Taverna et al. 2015 [24]	Urine	98	96,4	1/6
**Lung**	McCulloch et al. 2006 [9]	Breath	99	99	1/2
	Ehmann et al. 2012 [20]	Breath	71	93	1/6
	Walczak et al. 2012 [21]	Breath	79	78	1/2
	Amundsen et al. 2014 [23]	Breath	56–64	8–33	1/6
		Urine	64–74	25–29	1/6

In our study, age was a factor influencing the sensitivity and specificity of the test. Indeed, younger women had poor sensitivity and specificity compared to older women. This is consistent with the findings of the two studies by Willis et al. [8] and McCulloch [9]. A possible explanation of this finding is that ageing can induce changes in both the quality and quantity of VOC [25]. In fact, ageing can impact sweat production from eccrine sweat glands. Changes in sweat production may potentially affect the composition of volatile organic compounds (VOCs) emitted by the body, as sweat contains some of these compounds. Consequently, ageing can lead to alterations in the quality and quantity of emitted VOCs, which could explain some of the observed variations in age-related VOC profiles in the study.

Diabetes influenced the test sensitivity in our study. In fact, several disturbances of sweating may occur in diabetic individuals. Some may experience excessive sweating over the face, axillae and under the breasts (in females) due to the loss of distal sweating of the four limbs caused by the diabetic autonomic neuropathy [26, 27]. These sweating disorders may alter VOC related to cancer which would explain the slightly lower sensitivities among individuals with diabetes in our study.

Previous reports showed that the sweat ionic composition differs between normal and hypertensive subjects. In fact, essential hypertension is likely to be associated with altered cellular ionic regulation. Thus, hypertensive patients will have a higher pilocarpine induced sweat volume and sweat excretion. As a consequence, there will be less sodium in the sweat of patients with higher blood pressures [28]. In our study, hypertension was a factor influencing the sensitivity of this detection method. A better sensitivity was found in women without hypertension. Regarding the specificity of the test, hypertension was retained as a factor influencing the result on the multivariate analysis and not on the univariate analysis. Future research should focus more on assessing the dog’s behavior in the presence of other diseases or comorbidities.

The presence of a benign mastopathy influenced the test specificity in our study. This was also reported in a study by Willis et al. in which four dogs were trained to distinguish between urine samples from patients with transitional cell carcinoma and control group including benign urological diseases. Specificity was 92% when the dogs had to distinguish between urines from the case group and those from healthy volunteers, while it decreased down to 56% with control urine taken from individuals with benign urological diseases [8]. In 2014, Wang et al. studied the gaz profiles of metabolites in exhaled breath samples. The authors reported that approximately 400 of the analyzed metabolites were consistently detected in 50% of breast cancer samples and benign mastopathy samples. Thus, these common VOC might confuse the dog and reduce his detection accuracy [29]. To shed light into this aspect, future studies should consider including a variety of non- malignant disease into control groups.

As in other studies [20, 21], ‘hospital odour’ was a potential confounder for our test’s sensitivity. Indeed, a characteristic ‘hospital odour’, derived mainly from disinfection, may get into the odor samples and the dogs may become easily conditioned to such specific odor. Thus, samples from both patients and controls should be collected in a similar setting, outside the hospital to improve the test’s sensitivity.

Our statistical analysis did not provide support for any difference in dog’s performance relative to tumor size. Moreover, our study showed that early-stage breast carcinomas emit comparable scents to advanced tumors as we found comparable results in the two subsets. This data seems particularly relevant as it suggests that the specific cancer odour may be used to detect early stages of cancer which is the ultimate goal of using dogs for cancer screening. In the study of Kumar et al., comparison of early (stage I) and late stages (stages II and III) within the esophageal and gastric adenocarcinoma groups did not demonstrate any significant VOC differences [30] whereas Ehmann et al. [20] reported that advanced tumor UICC stage IV may impair the display accuracy of sniffer dogs. This could be explained by the presence of secondary lung tissue reactions such as inflammation and necrosis in advanced tumors.

In our study, for patients who performed the test during chemotherapy, the sensitivity of the test was significantly lower than for those who performed the test far from chemotherapy. It is likely that the completion of chemotherapy reduced the number of cancer cells in the patients’ bodies, thereby reducing the number of VOC emitted by the tumor.

We did not find any data in the literature on the effect of chemotherapy on the sensitivity of the detection test.

## Conclusion

To the best of our knowledge, the present study is the first to rely on this transcutaneous method to detect breast cancer by canine olfaction using a wide range of samples and including patients undergoing chemotherapy. This is also the first study to compare the accuracy of canine detection in breast cancer according to women and tumor characteristics.

One of the strengths of this method is its simplicity: the use of ordinary sampling tubes which can be easily handled by the sample donor without any special training and the test can be easily carried out at home in complete privacy. Moreover, this method is completely safe and harmless and women will only have to place a compress in contact with their breast(s). There is therefore no contact between the woman and the dog during the process of the test. We hope this work will raise public awareness of the cancer detection dogs and promote their practical use.

Due to the relative accessibility, simplicity and low cost of using dogs for cancer screening, this method would have good prospects, especially in low-income countries where common access to technologically advanced screening methods is still difficult. In fact, this method can be replicated abroad in low-resource countries without much equipment.

Future research should focus on developing optimal canine olfactory detection protocols that will be based on internationally standardized training methods and inclusion of more dogs to confirm reproductivity. Upcoming studies should seek to control for a greater number of potential confounding factor in order to identify which ones may influence the dog’s detection accuracy.

## Limitations

Although our results provide new insights into this field, the present study is subject to limitations. First, early enrollment of patients in the current study was sporadic, resulting in a limited number of skin samples available for training. Also, it took longer than expected to get enough samples to prepare for the final testing phase. As a result, training was spread out over a longer period We advise future studies to provide a sufficient number of samples to complete the study before training begins. Thus, a continuous system for recruiting cancer and control patients should be established to ensure that dogs have enough new specimens to maintain their performance level even after the study is completed.

Second, while the inclusion and exclusion criteria are appropriate for the study's objectives, it's important to acknowledge that these criteria may limit the generalizability of the findings to a broader population.

Moreover, we obtained these powerful results using only one dog. However, our results may not be reproducible with other types of dogs. The dog’s breed can influence the detection performance due to olfactory receptor polymorphisms [31]. It would be essential to expand established training methods to multiple dogs.

Another drawback is the environment in which we work. The conditions of the testing were not always optimal since training and testing occurred in an open-air environment. In fact, we had to interrupt the training phase because of the high temperatures during the months of July and August. Sometimes Roy was distracted by other females in heat in the training center and therefore his concentration was reduced. For future experiments, we recommend prepare a well-equipped room with a controlled temperature to any source of distraction for the dogs.

## Data Availability

The datasets generated during and/or analyzed during the current study are available from the corresponding author on reasonable request.
